# Input-dependent synaptic suppression by pregabalin in the central amygdala in male mice with inflammatory pain

**DOI:** 10.1016/j.ynpai.2021.100078

**Published:** 2021-11-18

**Authors:** Sumii Yamamoto, Yukari Takahashi, Fusao Kato

**Affiliations:** a Department of Anesthesiology, Faculty of Medicine, University of Tsukuba, Tsukuba, Ibaraki 305-8575, Japan; b Department of Neuroscience, The Jikei University School of Medicine, Minato, Tokyo 105-8461, Japan; c Center for Neuroscience of Pain, The Jikei University School of Medicine, Minato, Tokyo 105-8461, Japan

**Keywords:** EPSC, Excitatory postsynaptic current, VDCC, voltage-dependent Ca^2+^ channel, CeA, central nucleus of the amygdala, BLA, basolateral amygdala, PGB, pregabalin, LPB, parabrachial nucleus, PPR, paired-pulse ratio, CeC/L, capsular and lateral part of the central nucleus of the amygdala, Pregabalin, Central amygdala, Latent inflammatory pain, Formalin model, Synaptic transmission

## Abstract

Pregabalin (PGB) is a synthetic amino acid compound most widely prescribed for chronic peripheral and central neuropathic pain. PGB is a ligand for the α_2_δ_1_ subunit of voltage-dependent calcium channels, and its binding reduces neurotransmitter release and thus inhibits synaptic transmission. The central nucleus of the amygdala (CeA) is a kernel site for the enhanced nociception-emotion link in chronic pain. The nociceptive information is conveyed to the CeA via the following two pathways: 1) the pathway arising from the basolateral amygdala (BLA), which carries nociceptive information mediated by the thalamocortical system, and 2) that arising from the external part of the pontine lateral parabrachial nucleus (LPB), that forms the final route of the spino-parabrachio-amygdaloid pathway that conveys nociceptive information directly from the superficial layer of the spinal dorsal horn. We compared the effects of PGB on the excitatory postsynaptic currents of neurons in the right CeA in response to electrical stimulation of BLA and LPB pathways using the whole-cell patch-clamp technique. Inflammatory pain was induced by intraplantar injection of formalin solution at the left hind paw.

At eight hours post-formalin, PGB reduced EPSCs amplitude of the BLA-to-CeA synaptic transmission, accompanied by a significant increase in the PPR, suggesting a decreased release probability from the presynaptic terminals. In addition, these effects of PGB were only seen in inflammatory conditions. PGB did not affect the synaptic transmission at the LPB-to-CeA pathway, even in formalin-treated mice. These results suggest PGB improves not simply the aberrantly enhanced nociception but also various pain-associated cognitive and affective consequences in patients with chronic nociplastic pain.

## Highlights

• Pregabalin attenuated the synaptic transmission in the central amygdala.
• This effect was only observed in the latent inflammatory pain model.
• Only the basolateral to central amygdala pathway was affected.
• The potentiated inputs from the parabrachial nucleus were pregabalin-insensitive.

## Introduction

Chronic pain is defined as “pain that persists for more than three months” (Treede et al., 2019) and is classified as one of the most prevailing health problems worldwide. Among various approaches to mitigate chronic pain of diverse etiologies in human patients, pharmacotherapy is yet one of the standard tactics of the first choice. In particular, gabapentinoids, such as pregabalin (PGB), (S)-3-isobutyl-γ-aminobutyric acid, are one of the most widely used centrally acting analgesics for more than ten years for intractable chronic pain such as postherpetic neuralgia, fibromyalgia, and neuropathic pain (Derry et al., 2019, Dowell et al., 2016, Finnerup et al., 2015, Gewandter et al., 2014). It has been shown, in animal models of the chronic pain, that PGB and other gabapentinoids exert their analgesic effects through binding to the α_2_δ_1_ subunit of voltage-dependent calcium channels (VDCCs), which play essential roles in VDCC trafficking to the synaptic active zones and Ca^2+^-dependent transmitter release (Stahl et al., 2013). Furthermore, the absence of the analgesic effect of PGB in mice with an α_2_δ_1_ subunit variant lacking binding affinity to PGB has supported the notion that α_2_δ_1_ subunit is the central target of gabapentinoid analgesics (Field et al., 2006, Patel and Dickenson, 2016). Notably, it is well established that PGB has a potent analgesic effect on chronic sustained pain while its effect on acute pain is limited (Carley et al., 2021). This characteristic would imply that PGB might affect mechanisms involved not simply in the transmission of nociceptive signals but rather in the CNS mechanism underlying the establishment of chronic pain through nociplastic mechanisms (Fehrenbacher et al., 2003, Tanabe et al., 2008). In support of this, the appearance of the analgesic effect of PGB is latent in patients with chronic pain (Carley et al., 2021). Such particularity of the PGB effect should be considered to understand the mechanism of its analgesic effect in animal models and human patients with persistent chronic pain.

Recent advances in the neural mechanism of chronic pain pointed to a crucial involvement of the plastic changes in brain networks, particularly the limbic system and the brainstem, in various aspects of chronic pain in human patients and experimental animal models (Kuner and Flor, 2016, Thompson and Neugebauer, 2017, Vachon-Presseau et al., 2016).

For example, aberrantly increased activity levels of the amygdala and related structures characterize the long-lasting therapy-persistent back pain patients (Vachon-Presseau et al., 2016). Furthermore, in rodents, activation of the limbic system induced by pain or artificial manipulation augment nocifensive behaviors in rodents (Miyazawa et al., 2018, Sugimoto et al., 2021, Wilson et al., 2019). Of these structures, the central nucleus of the amygdala (CeA) is a site of interest because of the following properties: 1) it receives direct nociceptive information of spinal and trigeminal origins from the lateral parabrachial nucleus (LPB), 2) it also receives indirect and integrated nociceptive information from the basolateral amygdala (BLA), and 3) these synaptic inputs are potentiated various models of acute to long-term pain. Notably, it was recently reported that the excitation of the CeA neurons would determine pain-associated behaviors and experience of pain (Miyazawa et al., 2018, Sugimoto et al., 2021). Thus, it is assumed that drugs that affect synaptic transmission in the CeA would mitigate the pain through modifying components underlying the amygdala-mediated sensitization and the emotional/affective complication in chronic pain (Corder et al., 2019, Neugebauer et al., 2020).

The purpose of this study is to examine whether PGB affects the synaptic transmission in the CeA of animals of persistent pain. This hypothesis was based on the previously presented studies showing 1) the amygdala is one of the brain regions with moderate and high expression of mRNA for the α_2_δ_1_ gene (Cole et al., 2005), PGB isotope binding site (Bian et al., 2006), and α_2_δ_1_ protein (Taylor and Garrido, 2008), and 2) systemic injection of PGB reduces CeA spontaneous and stimulus-evoked neuronal activity in peripheral neuropathic animals (Gonçalves and Dickenson, 2012). In this study, we compared the effects of PGB on the excitatory synaptic inputs to CeA neurons of two distinct main origins, LPB and BLA (Miyazawa et al., 2018). Both of these two structures are activated in chronic pain models and involved in distinct aspects of pain-associated outcomes (Corder et al., 2019, Ikeda et al., 2007, Miyazawa et al., 2018, Neugebauer, 2015, Neugebauer et al., 2003) and the neurons in both of the LPB and BLA, the origins of these pathways, express α_2_δ_1_ (Bian et al., 2006, Cole et al., 2005, Taylor and Garrido, 2008). We compared the effects of PGB on these synaptic inputs in naïve or non-inflamed animals and those in animals with latent inflammatory pain.

## Materials and methods

The manipulation of the animals was approved by the Institutional Committee for the Care and Use of Experimental Animals of The Jikei University School of Medicine (Approval No. 2017-009). All animal experiments were conformed to the Guidelines for Proper Conduct of Animal Experiments of the Science Council of Japan (2006) and the guidelines of the International Association for the Study of Pain (Zimmermann 1983).

### Animals and formalin inflammatory pain model

Male 3–8 weeks-old C57BL/6 mice were purchased from Japan SLC Inc. (Hamamatsu, Japan) and housed on a 12-h light/dark cycle. Food and water were freely accessible. A formalin-induced inflammatory pain model was made by subcutaneous injection of 20 µL of 5% formalin; diluted 37% formaldehyde solution by saline (Nacalai Tesque Inc., Kyoto, Japan) into the plantar surface of the left hindpaw. For non-inflamed mice, an equal volume of saline was injected (“saline” group), or no injection was made (called as “naïve” group). Injections were made around midnight by an experienced co-author (Y.T.). At the time of injections, this injector knew the kind of the injecting solution (either containing formalin or saline) and confirmed the immediate emergence of typical licking and flinching behaviors in the formalin-injected animals and their absence in the saline-injected ones. All animals injected with formalin showed these specific nocifensive behaviors. The choice of the solution was randomized. About a few min later, the mouse was returned to the home cage, placed in a dark animal room and remained in the same home cage until the following day (∼08:00). Thus, we did not evaluate the first and second phase nocifensive behaviors except for that immediate post-injection observation. On the next day at 8:00, another experimenter took the animal without knowing the drug it had received on the previous night and prepared the slice. The treatments of the animals (formalin, saline, and naive) were randomly scheduled by one of the authors who did not participate in electrophysiological recordings so that these experiments were evenly performed.

### Preparation of slices

At around 8:00 in the morning, a mouse was taken from the home cage, anesthetized with isoflurane (5%), and sacrificed. Coronal brain slices, 400-μm thick, containing the central amygdala, were prepared using a vibrating blade slicer (Pro 7; Dosaka, Kyoto, Japan), transferred in an ice-cold cutting solution containing (in mM) KCl 2.5, CaCl_2_ 0.5, MgSO_4_ 10, NaH_2_PO_4_ 1.25, thiourea 2, sodium pyruvate 3, N-methyl-D-glucamine 93, HEPES 20, N-acetyl-L-cysteine 12, _D_-glucose 25, _L_-ascorbic acid 5, and NaHCO_3_ 30, equilibrated with 95% O_2_ + 5% CO_2_ (osmolality, ∼290 mOsmol/kg H_2_O) at 34 °C for 15 min. Slices were moved to the ACSF (in mM) NaCl 125, KCl 3, CaCl_2_ 0.1, MgCl_2_ 5, NaH_2_PO_4_ 1.25, _D_-glucose 10, _L_-ascorbic acid 0.4 and NaHCO_3_ 25 (pH 7.4) equilibrated with 95% O_2_ + 5% CO_2_, then maintained for several hours in ACSF at room temperature.

### Electrophysiological recordings

Neurons in the capsular and lateral part of the CeA (CeC and CeL; “CeC/L” in this study) were visually identified using oblique illumination optics microscopy (BX51WI, Olympus) and a charge-coupled device camera (IR-1000, DageMTI). Whole-cell recordings were made from brain slices in a recording chamber continuously perfused with oxygenated ACSF (95% O_2_/5% CO_2_) at a 1.5–2.0 ml/min flow rate. The patch-clamp electrodes were made from borosilicate glass pipettes (1B150F-4; World Precision Instruments, Sarasota, FL). The tip resistance of the recording electrodes was 5–10 MΩ, and the recording electrodes were filled with internal solution containing (in mM) K-gluconate 125, NaCl 6, HEPES 10, Na_2_-phosphocreatine 12, ethylene glycol-bis (2-aminoethylether) -N,N,N’,N’-tetraacetic acid (EGTA) 5, CaCl_2_ 1, MgCl_2_ 2, MgATP 2, QX-314 5, and Mg guanosine 5′-triphosphate (GTP) 0.5 (pH 7.3; osmolarity, 290–310 mOsmol/kg H_2_O). EPSCs were recorded at a holding potential of −70 mV with a patch-clamp amplifier (Axopatch 200B, Molecular Devices, Sunnyvale, CA), low-pass filtered at 2 kHz, and sampled at 10 kHz at a 16-bit resolution with a PowerLab interface (ADInstruments, Sydney, Australia) and pClamp 10 software (Molecular Devices, Sunnyvale, CA). Liquid junction potential was not compensated.

## LPB and BLA afferent pathways stimulation

**Fig. 1:**
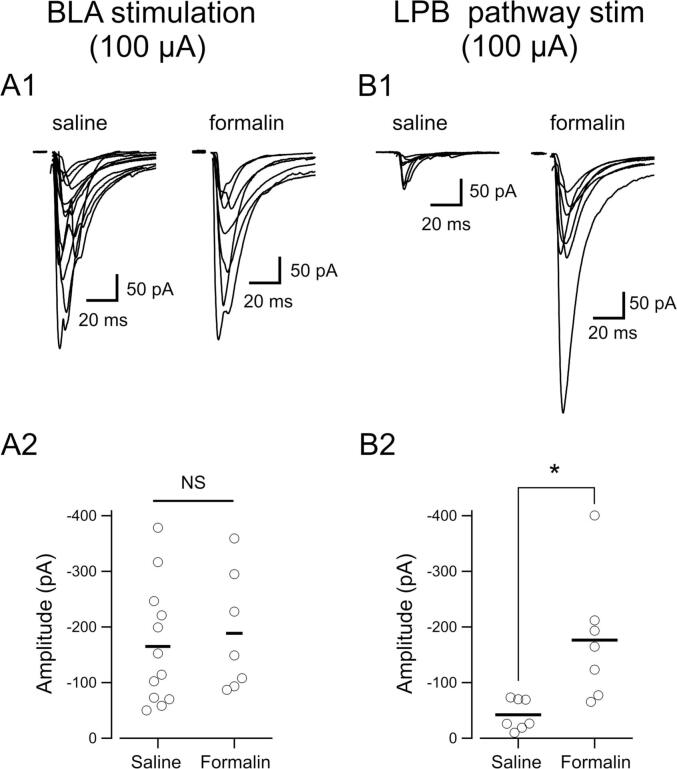
Effect of saline- and formalin- intraplantar injection on EPSC_BLA_ (EPSC evoked by BLA stimulation, left panels) and EPSC_LPB_ (by LPB pathway stimulation, right panels) recorded in CeC/L neurons. A1 and B1 representative overlaid traces (mean of the responses to 8 consecutive stimuli) of EPSC_BLA_ and EPSC_LPB_, respectively, recorded in CeC/L neurons from mice receiving prior saline or formalin injection to the hind paw. A2 and B2 summarize the mean EPSC amplitude evoked by constant intensity (100 µA) stimulation of the BLA and LPB pathway. The numbers of neurons are 12 (saline in A1), 7 (formalin in A1), 7 (saline in B1), and 7 (formalin in B1). Each circle represents the average amplitude from each neuron, and the horizontal bars indicate the mean value of each group.

EPSCs were evoked in CeC/L neurons by electrical stimulation of the two afferent fibers targeting the CeC/L: the LPB and BLA pathways. We used custom-designed bipolar parallel stimulation electrodes (TOG211-039a, Unique Medical Co., Ltd., Tokyo, Japan) under microscopic control as described previously reported (Watabe et al., 2013). BLA-stimulating electrode was placed in the ventral BLA near the borderline to the CeA (Fig. 5B). The EPSC evoked by stimulation of this electrode location was denoted as EPSC_BLA_. LPB-stimulating electrode was placed onto the fibers that run dorsomedial to the CeA and ventral to, but outside of, the caudate-putamen (Fig. 5B). The EPSC evoked by stimulation of this pathway was denoted as EPSC_LPB_. The stimulation intensity was controlled using a constant current mode of an ISO-flex isolator (A.M.P.I, Israel) connected to Master-8 (A.M.P.I, Israel). The stimulation intensity was optimized at a fixed constant current in a range of 20 µA–200 µA for evoking EPSC_BLA_ and 40 µA–500 µA for evoking EPSC_LPB_, so that the average amplitude of EPSC over trials would be around 100 pA. Only in Fig. 1, in which the amplitudes of EPSC_LPB_ and EPSC_BLA_ were compared between saline- and formalin-treated groups, the stimulation intensity was fixed at 100 µA. The stimulation intensity was fixed for the recording from a neuron except explicitly written. The changes in series resistance were monitored using the responses to pre-pulse current injections made regularly immediately after the stimulation pulse, and data were discarded if they varied more than 20% within an experiment. The paired-pulse ratio (PPR) of EPSCs was calculated as the ratio of the amplitudes of successive two ESPCs (EPSC_2nd_/EPSC_1st_.) evoked by two pulses with an interstimulus interval of 100 ms as All experiments were carried out at room temperature (20–25 °C).

### Drugs and their application

Pregabalin was purchased from Sigma (St. Louis, MO). A stock solution of PGB at 100 mM in MilliQ water was made and frozen until the day of use. The solution at the final concentration was made on the day of use by resolving with fresh carbogen-saturated ACSF at a concentration of 100 µM.

### Data and statistical analysis

The recorded membrane current was analyzed offline using an Igor Pro 7 or Igor 8 (WaveMetrics, Lake Oswego, OR) using macros written by one of the authors (F.K.). Peak amplitude was measured based on the averaged waveform of evoked EPSCs (eight consecutive trials). Values are expressed as mean ± standard error of the mean (SEM). Statistical analysis consisted of Student's *t*-test, Kruskal-Wallis test, Friedman test, Wilcoxon signed-rank test, and estimation statistics.

## Results

We have already demonstrated that intraplantar or orofacial injection of formalin causes latent potentiation of the synaptic transmission between the fibers of LPB origin and CeC/L neurons (Miyazawa et al., 2018, Shinohara et al., 2017, Sugimura et al., 2016). This increase was accompanied by a marked increase in the number of neurons expressing c-Fos in the LPB, but to a much smaller extent in the BLA (Miyazawa et al., 2018). Therefore, we first compared the amplitudes of EPSC_BLA_ and EPSC_LPB_ in slices from saline-injected and formalin-injected mice (Fig. 1). The intraplantar injection of formalin resulted in typical nocifensive behaviors composed of licking and flinching of the hind paw. The brain slices were prepared at approximately 8 h after the formalin or saline injection, at which the mice showed apparently normal behaviors as before the injections. Stimulation of BLA (by the electrode placed in the BLA) and that of LPB pathway (by the electrode placed on the putative LPB-CeA fibers) triggered short- and stable-latency EPSCs in the neurons recorded at − 70 mV (Fig. 1A and 1B) with varied amplitude depending on the stimulation pathways and individual neurons. In response to stimuli of the same intensity (100 µA), the amplitude of EPSC_BLA_ did not significantly differ between the formalin- and saline-treated mice (Fig. 1A1 and 1A2; P = 1.000, Kruskal-Wallis test with Bonferroni correction; n = 12 and 7, for saline and formalin), while the EPSC_LPB_ amplitude was significantly larger in formalin-treated mice (Fig. 1B1 and 1B2; P = 0.029, Kruskal-Wallis test with Bonferroni correction; n = 7 and 7, for saline and formalin, respectively) suggesting that, unlike the synaptic transmission from the LPB to CeA, that from the BLA is not affected by persistent inflammatory pain.

### PGB decreased the amplitude of EPSC evoked by BLA stimulation only in formalin-treated mice

**Fig. 2:**
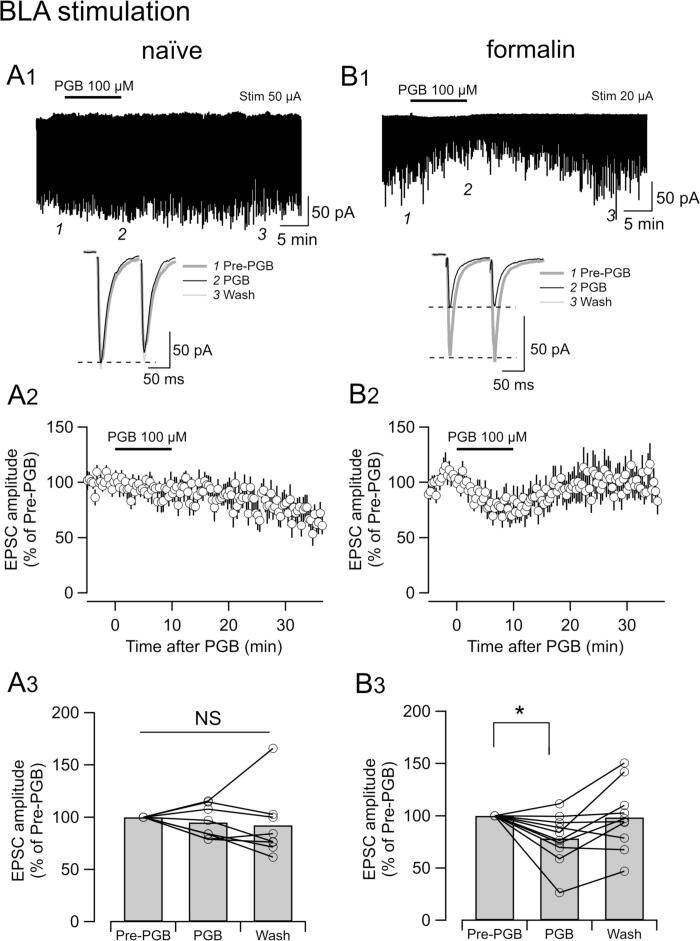
The effects of PGB on BLA-to-CeC/L synaptic transmission. Representative traces of EPSCs in naïve mice (A1, stimulation intensity; 50 µA) and formalin-treated mice (B1, stimulation intensity; 20 µA). PGB (100 µM) was applied for 10 min at the time indicated by the bar in top traces of Fig. 2A1 and 2B1. Averaged traces (bottom in Fig. 2A1 and 2B1) of eight consecutive EPSCs evoked 1; just before the application of PGB (Pre-PGB), 2; just before the discontinuation of PGB (PGB), and 3; more than 20 min after wash (Wash), which were sampled at respective time points indicated in top traces of Fig. 2A1 and 2B1. Broken lines indicate the level of the peak of the first EPSCs. The time course of the first peak amplitude normalized by the Pre-PGB amplitude in naïve mice (A2) and formalin-treated mice (B2). Mean ± SEM. Average values of EPSC amplitude normalized by the “Pre-PGB” value from naïve (A3, n = 8) and formalin (B3, n = 10) mice. Line and circles indicate each cell. Bars are the average. NS, not significantly different. *P < 0.05 after Bonferroni correction.

We then examined the effects of PGB (100 µM) on EPSC_BLA_ and EPSC_LPB_ in brain slices from mice that received an intraplantar injection of formalin, saline or without injection (naïve) at approximately 8 h before the slice preparation. First, we analyzed the effects of PGB on EPSC_BLA_ (Fig. 2). While PGB did not exert apparent changes in the EPSC_BLA_ in the CeC/L neurons from naïve mice (Fig. 2A1 and 2A2), it markedly decreased the amplitude of EPSC_BLA_ in the formalin-injected group (Fig. 2B1 and 2B2). This effect was initiated immediately after the beginning of the application and recovered almost to a pre-application level after the washout (Fig. 2B2).

PGB significantly decreased EPSC_BLA_ amplitude to 78.15 ± 7.52% of pre-PGB values after 10-min application (P = 0.022; Friedman test with Bonferroni correction), which recovered to 98.30 ± 9.88% after greater than 20-min washout (n = 10 neurons from seven slices from three mice; Fig. 2B2 and 2B3). This decrease in the EPSC_BLA_ amplitude by PGB was significant after 10-min application (78.15 ± 7.52% and 98.30 ± 9.88% of Pre-PGB amplitude for during PGB and wash, respectively; n = 10). Friedman test showed a significant difference in EPSC_BLA_ amplitude between Pre-PGB and PGB (P = 0.022 after Bonferroni correction; Fig. 2B3, 7 slices from three mice, n = 10). There was no significant difference in EPSC_BLA_ amplitude between pre-PGB and wash (Fig. 2B3). In contrast, EPSC_BLA_ was not significantly affected by PGB in mice without prior treatment with formalin (Fig. 2A3, 5 slices from two mice, n = 8, P = 0.197, Friedman test).

In the recordings in Fig. 2, we evoked EPSC_BLA_ with stimulus at intensities of 20 µA–140 µA (69.4 µA ± 19.6 µA; n = 9) and 25 µA–200 µA (62.9 ± 16.4 µA; n = 7), for neurons from formalin-treated and naïve mice, respectively (the values for a neuron in each of two groups were not registered non-intentionally). These intensities were adjusted to obtain a similar amplitude of EPSC_BLA_ between 100 µA and 200 µA before PGB application over the neurons. There was no significant difference between the stimulation intensity for these experimental conditions (P = 0.791, unpaired *t*-test; df = 14). These results demonstrate that PGB attenuates synaptic transmission from the BLA to CeA neurons in the slices prepared from mice with persistent inflammatory pain.

To confirm that this significant difference between saline- and formalin-treated mice in the inhibitory effect of PGB on EPSC_BLA_ amplitude shown above did not result from the different stimulation intensities used in each neuron recording, we compared the effects of PGB on EPSC_BLA_ evoked by 50-µA and 100-µA stimulation intensities in slices from other cohorts of mice. PGB reduced the EPSC_BLA_ amplitude to 43.8% ± 10.0% (50-µA stimulation; P = 0.018, Wilcoxon signed-rank test) and to 52.2% ± 9.8% (100-µA stimulation) of Pre-PGB values (P = 0.018; seven neurons from seven slices from four mice) in formalin group, and to 86.8 ± 17.6% (50-µA stimulation; P = 0.510) and 77.0% ± 11.1% (100-µA stimulation; P = 0.074; 13 slices from seven mice, n = 14) in saline group. We found no significant difference between the effects of PGB between the 50-µA and 100-µA stimulation intensities in both groups (P = 0.331 and P = 0.128, Wilcoxon signed-rank test, saline and formalin groups, respectively).

### The decrease in EPSC_BLA_ amplitude by PGB in formalin-treated mice was accompanied by an increase in the paired-pulse ratio

**Fig. 3:**
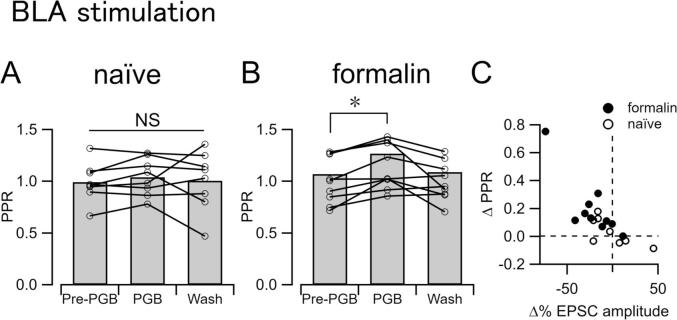
An increase in the paired-pulse ratio accompanied the decrease in EPSC_BLA_ amplitude. The averaged paired-pulse ratio of pre-PGB, PGB, and wash in naïve mice (A, n = 8) and formalin-treated mice (B, n = 10). Lines and circles indicate values from each neuron. Bars are average. *P < 0.05, analyzed with Friedman test with Bonferroni correction. (C) Correlation between the normalized changes of amplitude with Pre-PGB (abscissa) and the changes of PPR (ordinate) in naïve (open circles; n = 8) and formalin mice (filled circles; n = 10).

The amplitude of EPSC_BLA_ caused by the second stimulation was larger than that caused by the first one before PGB application (pre-PGB), which became almost identical during application of PGB (Fig. 2B1, bottom). In most of the CeC/L neurons recorded from formalin-injected mice, the PPR was increased by PGB (1.07 ± 0.09, 1.27 ± 0.14, and 1.08 ± 0.10 for before (Pre-PGB), during (PGB) and wash, respectively; n = 10). Friedman test showed a significant difference in the mean between Pre-PGB and PGB (P = 0.011 after Bonferroni correction; Fig. 3B, seven slices from three mice, n = 10) in a manner negatively correlated with the changes in ESPC_BLA_ amplitude (filled circles in Fig. 3C; r =  − 0.758; Spearman's rho; P = 0.011). These significant changes in the PPR and the amplitude-PPR correlations were not observed in naïve mice (PPR, 0.99 ± 0.07, 1.04 ± 0.06 and 1.00 ± 0.10 for pre-PGB, PGB and wash, respectively; n = 8; P = 0.687; Friedman test; Fig. 3A) (open circles in Fig. 3C; r =  − 0.595; Spearman’s rho; P = 0.12). These results suggest that PGB attenuates the synaptic transmission from the BLA to the CeA mostly through affecting the presynaptic release probability at the axon terminals of the BLA origins.

### Synaptic transmission from the LPB pathway to the CeC/L neurons was not affected by PGB regardless of the formalin-induced inflammation

**Fig. 4:**
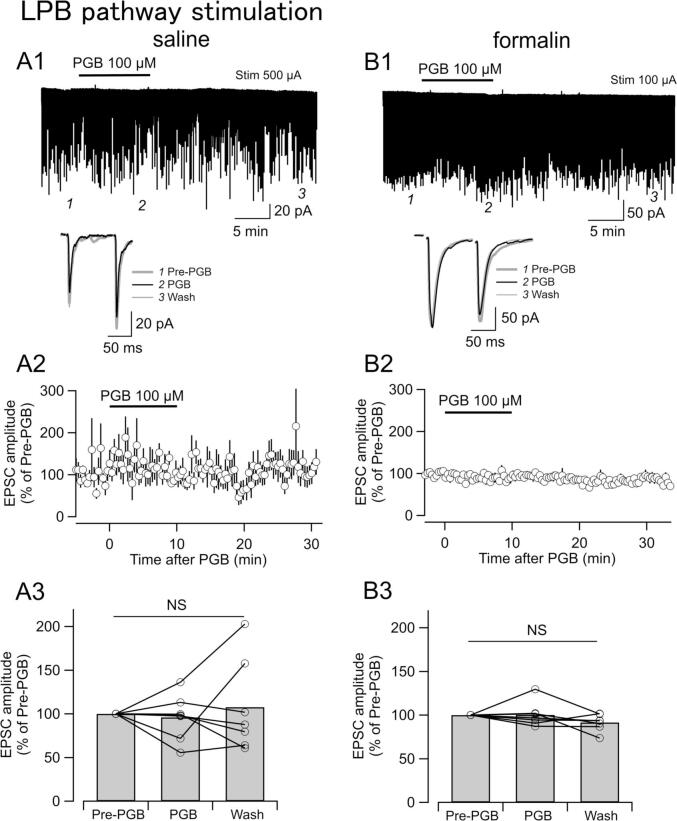
The effects of PBG on EPSC_LPB_ amplitudes in CeC/L neurons. Representative traces of the amplitudes of EPSCs in naïve mice (A1, stimulation intensity; 500 µA) and formalin-treated mice (B1, stimulation intensity; 100 µA) PGB (100 μM) was applied for 10 min at the time indicated by the bar in top traces of Fig. 4A1 and 4B1. Averaged traces (bottom in Fig. 4A1 and 4B1) of eight consecutive EPSCs evoked 1; Pre-PGB, 2; PGB, and 3; Wash, which were sampled at the time points indicated in top traces of Fig. 4A1 and 4B1. Broken lines indicate the level of the peak of the first EPSCs. The time course of the first peak amplitude normalized by Pre-PGB amplitude in saline (A2) and formalin (B2) mice. Mean ± SEM. Average values of EPSC amplitude normalized by the “Pre-PGB” value from naïve in saline (A3, n = 7) and formalin (B3, n = 7) mice. Circles indicate the individual ratio for each neuron. Line and circles indicate each cell. Bars are the average. NS, not significantly different (with Friedman test).

In contrast to the attenuation of EPSC_BLA_ by PGB in the brain slices from inflamed mice, we failed to find significant changes in the amplitude of EPSC_LPB_ in response to 10-min application of PGB at a concentration (100 µM) that significantly affected EPSC_BLA_ amplitude in the slice prepared from inflamed mice (Fig. 4; see Fig. 2B). This absence of the effect of PGB on EPSC_LPB_ was observed regardless of prior formalin injection (Fig. 4B3; n = 7, P = 0.18 in formalin-treated; n = 7; P = 0.65 in saline-treated preparation; Friedman test). In this series of experiments, the stimulus intensity was adjusted so that the amplitude of EPSC_LPB_ gave values between 100 pA and 200 pA (100 µA for formalin-treated and 100–500 µA for saline-treated mice) for the comparison of the relative changes in the EPSC_LPB_ amplitude between formalin- (100.26 ± 5.28% and 91.54 ± 3.62% of Pre-PGB amplitude for during (PGB) and wash, respectively; Fig. 4B3, 6 slices from three mice, n = 7, P = 0.180, Friedman test) and saline-treated mice (95.98 ± 9.95% and 107.78 ± 20.06% of Pre-PGB for during (PGB) and wash, respectively; Fig. 4A3, 4 slices from three mice, n = 7, P = 0.651, Friedman test). These results indicate that PGB exerts only limited effects on the LPB to CeC/L transmission regardless of the presence of inflammation that itself potently affects this transmission.

**Fig. 5:**
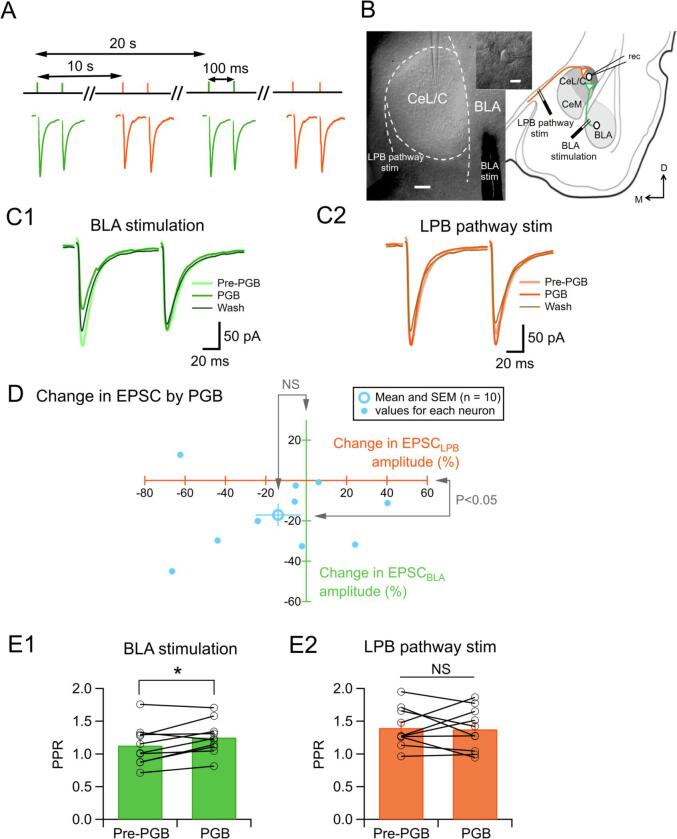
Effect of PGB on EPSC_BLA_ and EPSC_LPB_ recorded in single CeC/L neurons of formalin-injected mice. Experimental design for alternating stimulation of pathways arising from the BLA and LPB converging to the CeC/L pathways (A). Representative video microscope images showing the electrode placement (B, left) and CeC/L recorded cell (B, upper middle) and the schema showing the recording configuration for EPSC_BLA_ and EPSC_LPB_ (B, right). Broken lines in B (left) indicate the definition for the zone of the CeC/L. Scale bars are 100 μm (B, left) and 10 μm (B, upper middle). Typical average traces of eight consecutive EPSC_BLA_ (C1, traces in green) and EPSC_LPB_ (C2, traces in orange) waveforms. The traces in light color, medium color and dark color indicate average traces for evoked “Pre-PGB”, “PGB, and “Wash”, respectively in terms of the application of PGB. Summary of the effects of PGB on the EPSC amplitude (D). The horizontal axis (orange), the change in EPSC_LPB_ amplitude (relative to pre-PGB value); the vertical axis (green), the changes in EPSC_BLA_ amplitude recorded in the same CeC/L neurons (shown with small light-blue filled circles; n = 10). The open light-blue circle represents the mean values with SEM. NS, not significantly different (D). Summary of the effect of PGB on the PPR of EPSC_BLA_ (E1, P = 0.026, n = 10) and EPSC_LPB_ (E2, P = 0.817, n = 10). Line and circles indicate each cell. Bars are the average. *P < 0.05. NS, not significantly different. (For interpretation of the references to color in this figure legend, the reader is referred to the web version of this article.)

### PGB affected EPSC_BLA_ but not EPSC_LPB_ recorded in the same CeC/L neurons

The above results suggest that PGB exerts its inhibitory effect on the synaptic transmission through affecting presynaptic mechanisms only at the axon terminals of the BLA neurons synapsing to the CeC/L neurons in the animals with inflammatory pain. As it has been shown that inputs of the LPB and the BLA origins converge to a large portion of CeC/L neurons (Miyazawa et al., 2018, Sugimura et al., 2016), we then compared the effects of PGB on the EPSC_BLA_ and EPSC_LPB_ recorded in single CeC/L neurons prepared from mice with a prior injection of formalin to confirm this specific effect of PGB on the axons of BLA origin. We placed two stimulation electrodes on the pathway of LPB origin and the BLA (Fig. 5B) and alternately stimulated these pathways at an interval of 20 s (10 s between the stimulation of each pathway; Fig. 5A). The stimulation intensity for each pathway was separately controlled so that both EPSC_BLA_ and EPSC_LPB_ gave values between 100 and 200 pA before the application of PGB (100 µM; Fig. 5C1 and C2).

Fig. 5D indicates the effect of PGB on the relative changes in amplitudes of EPSC_BLA_ (Y-axis in green) and EPSC_LPB_ (X-axis in orange) recorded in a single neuron. While EPSC_LPB_ showed varied and inconsistent responses to PGB (X-axis locations of small light-blue-filled circles in Fig. 5D), EPSC_BLA_ was decreased by PGB in most neurons regardless of the changes in EPSC_LPB_ amplitude recorded in the same neuron (Y-axis locations of small light-blue-filled circles in Fig. 5D). Though EPSC_LPB_ was not significantly affected by PGB (P = 0.203, Wilcoxon signed-rank test; n = 10), EPSC_BLA_ was significantly reduced by PGB (P = 0.022, Wilcoxon signed-rank test; n = 10; Open light-blue circle in Fig. 5D). A significant increase in PPR accompanied this decrease in EPSC_BLA_ amplitude by PGB (Fig. 5E1; P = 0.026, paired *t*-test; n = 10), but not for the EPSC_LPB_ (Fig. 5E2; P = 0.817, paired *t*-test; n = 10).

## Discussion

The gabapentinoids are the most frequently subscribed drugs against chronic pain. PGB, the most commonly prescribed medication of the analgesic gabapentinoids, is well characterized to exert its analgesic effect by binding to α_2_δ_1_ subunits of VDCCs (Field et al., 2006, Patel and Dickenson, 2016, Stahl et al., 2013). Indeed, PGB attenuates the synaptic transmission from the primary afferents to the dorsal horn neuron in the neuropathic pain model of rodents (Matsuzawa et al., 2014), where the expression of α_2_δ_1_ subunits are dense. However, despite rich expression of the α_2_δ_1_ subunits also in some of the brain regions and the established clinical efficacy of PGB in the cohort of chronic pain patients with central sensitization, such as the fibromyalgia (Arnold, 2017), it remains undetermined whether PGB affects pain-associated activities in the brain, particularly in the regions involved in the expression of nociplastic pain.

Using membrane current recording from neurons in the CeA from brain slices prepared from mice after formalin or saline injection, we demonstrated 1) PGB attenuated excitatory synaptic transmission from the BLA to the CeA in the mice with inflammatory pain, 2) this effect was not observed in mice without inflammatory pain, 3) this attenuation by PGB of the BLA to CeA transmission involved changes in the presynaptic release probability, 4) in contrast, PGB did not affect the excitatory synaptic transmission from the LPB to the CeA regardless of the presence of inflammatory pain, despite the markedly augmented synaptic transmission to CeA observed only in inflamed animals. Thus, this is the first to demonstrate an inhibitory effect of PGB on excitatory synaptic inputs to the CeA neurons, which depended on the input pathway and inflammatory pain state.

### PGB inhibits inputs from BLA

α_2_δ_1_ subunits of VDCC are widely expressed in various brain regions. Of these, the BLA is a site that expresses a high density of α_2_δ_1_ subunit mRNAs (Cole et al., 2005), α_2_δ_1_ protein (Taylor and Garrido, 2008), and [^3^H]-PGB binding (Bian et al., 2006), suggesting PGB can bind to these α_2_δ_1_ subunits in the BLA and modulate its function. The present results showing an inhibitory effect of PGB on the excitatory transmission from the BLA to the CeC/L neurons might have resulted from such direct binding of PGB to α_2_δ_1_ subunits expressed at the synaptic terminals of the fibers arising from the BLA in the CeA. The increase in the PPR (suggestive of decreased release probability), which accompanied the decrease in EPSC_BLA_, also suggests the presynaptic site of action of the PGB effect. This result would indicate that the α_2_δ_1_ subunit molecules expressed in the CeA-projecting BLA neurons are trafficked to the axon terminals, where they regulate the BLA-to-CeA synaptic transmission.

It is established that the expression of α_2_δ_1_ subunits is increased in dorsal root ganglion (DRG) and the superficial layer of the spinal dorsal horn of rats/mice with peripheral nerve injury (Luo et al., 2018), suggesting that aberrantly augmented activities in the peripheral nociceptive afferents can upregulate the quantity and function of α_2_δ_1_ subunits at the synapses transmitting nociceptive information (Boroujerdi et al., 2008). Therefore, a plausible and widely accepted scenario is that PGB binds to such increased α_2_δ_1_ subunits at the primary afferent terminals to attenuate glutamate release and decrease nociceptive sensitivity (Matsuzawa et al., 2014). However, such changes in the α_2_δ_1_ subunit expression in the spinal cord would not simply favor the formalin-dependent appearance of significant effect of PGB at the BLA to CeA synapses in slices isolated from the lower brain and spinal cord. Instead, it is well expected that the augmented expression level of the α_2_δ_1_ in the central pain pathways in response to sustained pain underlies the effect of PGB in the CeA as suggested by Nasca et al., who reported that a trimethylthiazoline exposure upregulates expression α_2_δ_1_ in an unidentified subregion of the amygdala (Nasca et al., 2013).

It remains unexplored how the expression level of α_2_δ_1_ subunits is regulated. Simple speculation is that the transcription factor activation in response to increased neuronal activities, such as those downstream of c-Fos, would play roles in increasing α_2_δ_1_ subunit expression. Indeed, at 3 h after orofacial formalin injection, c-Fos expression is robustly increased in the right CeA and bilateral LPB (Miyazawa et al., 2018, Touj et al., 2019). However, the expression of c-Fos in the BLA was limited or slight in these models, suggesting that the increased α_2_δ_1_ subunit expression does not fully account for the BLA-only effect of the PGB in this study. PGB also affects the trafficking of the α_2_δ_1_ subunit from the soma (Bauer et al., 2009). It remains to be elucidated whether increased neuronal activity in the soma causes increased trafficking of α_2_δ_1_ subunit to the axon terminal. If this is the case, it would be likely that such pain-associated activation of the BLA neurons gave rise to increased membrane expression of α_2_δ_1_ in the terminals of the axon projecting to the CeA and caused a more potent decrease in EPSC_BLA_ in the CeA by PGB in the amygdala of formalin-treated animals, but not in naïve or saline-treated animals. This possibility requires experimental examination in future studies.

### PGB effect on EPSC_BLA_ was only observed in mice with inflammatory pain

The present results indicate that the inhibitory effect of PGB is only observed in animals with inflammatory pain. As an expression of α_2_δ_1_ subunits has been shown in the BLA of naïve animals (Bian et al., 2006, Cole et al., 2005, Taylor and Garrido, 2008), our results would mean that α_2_δ_1_ subunits already expressed in naïve animals are not functional or not numerous enough in the presynaptic terminals of the BLA-to-CeA synapses of naïve animals to express the synaptic effect of PGB. Indeed, increased expression of α_2_δ_1_ in the spinal dorsal horn or dorsal root ganglion has been documented in a large variety of animal models of pain and human patients with neuropathy or central sensitization (Tuchman et al., 2010). For example, nerve injury-induced up-regulation of the α_2_δ_1_ subunit in the dorsal horn is a key factor for gabapentinoid analgesia (Bauer et al., 2009, Luo et al., 2018). The most plausible interpretation of the present results would be that the formalin injection increased α_2_δ_1_ subunit expression in the spinal cord and the BLA to reach a level sufficient for effective presynaptic inhibition by PGB. These results imply that inflammatory pain has boosted the expression of α_2_δ_1_ subunits at the presynaptic terminals of the BLA-CeA synapses. Therefore, it is essential in future studies to evaluate whether expression level and subcellular localization of α_2_δ_1_ subunits are affected in the BLA of the animals with persistent pain and peripheral inflammation. It would also be necessary to identify the molecular mechanisms underlying the nociceptive activity-dependent upregulation of α_2_δ_1_ molecules, as well as their trafficking, in the brain structures other than the spinal cord, including the central amygdala.

### PGB did not affect LPB-to-CeA transmission despite its potentiation in inflamed mice

In contrast, we failed to observe such an inhibitory effect of PGB on the excitatory synaptic transmission from the fibers arising from the LPB to the CeA neurons. This result was unexpected because we have demonstrated that the excitatory synaptic transmission at the LPB-CeA synapses is robustly potentiated in the animals with formalin-induced inflammatory pain (Miyazawa et al., 2018, Shinohara et al., 2017), which was also confirmed in this study (Fig. 1). Thus, the molecular mechanism underlying this synaptic potentiation is likely independent of the altered expression level of α_2_δ_1_ subunits. The most straightforward interpretation would be that the LPB does not express α_2_δ_1_ messengers, unlike the BLA. However, this is not likely the case. Cole et al. demonstrate rich expression of α_2_δ_1_ mRNA in the rat LPB (Cole et al., 2005), and the Allen mouse brain database also shows a high expression level of α_2_δ_1_ mRNA in the LPB region (Experiment number 72119649 and 75042246; https://mouse.brain-map.org/). Therefore, a plausible interpretation is that, at the LPB-to-CeC/L synapses, the expression of α_2_δ_1_ subunits is not so elevated as to be affected by PGB in the formalin-treated mice. There are two possibilities for this to be the case. First, due to undetermined mechanisms, α_2_δ_1_ subunits are not expressed on the presynaptic membrane of LPB-to-CeC/L synapses, unlike the BLA-CeC/L synapses, in both naïve and inflamed animals. Target-dependent sorting and trafficking of presynaptic molecules have been documented in other synapses (Éltes et al., 2017, Yamamoto et al., 2010), supporting this possibility. Second, as we have measured the effect of PGB at a single time point, i.e., 8 h post-formalin, it is possible that upregulated expression of α_2_δ_1_ subunits in the soma of LPB neurons, if any, did not yet influence the effect of PGB at the LPB-CeA synapses simply because of the difference in the distances from the BLA to CeA and that from the LPB to CeA. Electron microscopic immunohistochemistry of the α2δ subunit proteins at the axon terminals arising from the BLA and LPB at various time points would provide direct morphological support for this interpretation, which remains technically challenging.

In this study, we observed acute PGB effects on synaptic transmission in the mice with latent inflammatory pain and found differences between distinct synapses. Hendrich et al. (2012) demonstrated in co-cultured DRG and dorsal horn (DH) neurons that the long-lasting (40–48 h) presence of PGB in the culture medium attenuates capsaicin-induced activation of excitatory transmission, suggesting that chronic PGB application would inhibit trafficking of VDCC complex to the synaptic terminals (Hendrich et al., 2012). If this situation also occurs in the BLA and LPB neurons expressing α2δ subunits, it would be expected that sustained administration of PGB in vivo throughout the entire span of the inflammation would reduce the terminal expression of α2δ subunit-containing VDCCs. This possibility should be tested in future studies. On the contrary, it would be interesting to examine whether PGB can attenuate the LPB-to-CeA transmission at later stages after formalin injection. As a sum, the balance between the inhibitory effect on the toward-terminal trafficking of the VDCC complex and synaptic inhibition at the terminals with trafficked PGB-sensitive VDCCs would determine the spatiotemporal phenotype of the PGB effect in sustained-pain in vivo. In contrast, the specific synaptic potentiation at the LPB to CeA synapses (Miyazawa et al., 2018) would be mediated by a synaptic mechanism distinct from that underlies the α_2_δ_1-_mediated synaptic suppression.

In this previous work made using rats (Miyazawa et al., 2018), we reported that activation of the right-side CeA was more correlated with the BLA activity than with the LPB activity at 3 h post-formalin, suggesting that the BLA might play a key role in determining the CeA neuronal activity in inflamed animals. In contrast to the bilateral and inflammation-side-dependent activation of the LPB, unilateral activation of the right-side CeA, regardless of the inflammation side, is sufficient for the widespread sensitization with the orofacial inflammation model (Sugimoto et al., 2021). The role played by the link among the bilateral LPB, BLA and CeA in determining the widespread sensitization in this latent inflammatory pain model should be addressed in future studies.

## Functional consequences

Accumulated lines of evidence from clinical and preclinical studies point to an essential involvement of the amygdala, particularly the CeA, in the establishment and maintenance of chronic pain (Bingel et al., 2002, Kato et al., 2018, Simons et al., 2014, Sugimoto et al., 2021, Thompson and Neugebauer, 2017, Vachon-Presseau et al., 2016, Wilson et al., 2019).

The roles of the central amygdala in chronic pain reported so far include enhanced nociception-emotion link, aberrant descending pain control, and associated expression of autonomic and endocrine responses (Simons et al., 2014). The present results provide a novel possibility that the PGB exerts its “analgesic” effects through affecting the synaptic excitability of the CeA neurons, in addition to the conventionally understood periphery-to-the spinal cord inhibition. As pain is “an unpleasant sensory and emotional experience” (Raja et al., 2020), inhibition by PGB of the synaptic excitation of CeA neurons, which are involved in many dimensions of pain, would have a broad and potent impact on controlling the experience of pain in patients with chronic pain. However, the mitigation of chronic pain symptoms by PGB is not necessarily universal in all types of chronic pain patients (McAnally et al., 2020). In parallel, the amygdala's involvement in the expression of pain depends on the time course and type of chronic pain (Hashmi et al., 2013). Evaluation of the degree to which the amygdala plasticity underlies the chronic pain experience of each patient at each timing within the progress of chronic pain would further improve the efficacy of the gabapentinoids.

## Limitations

The following issues remain to be addressed in future studies. 1) The effect of PGB on the CeA neurons in female animals. The present study is based on the data collected only from male mice. It is important to note that increasing lines of evidence propose different molecular/cellular mechanisms for the expression of pain-associated behaviors between male and female animals (Sorge et al., 2015). A recent report demonstrated that, also in the amygdala, different sets of molecules are recruited in male and female mice with plantar incision-induced pain (Baptista-de-Souza et al., 2020). Furthermore, the mode of action of the analgesic effect of PGB depends on the sex of animals in different pain models (Ungard et al., 2020). Therefore, it is possible the findings in this study made in male mice concerning the effect of PGB on BLA and CeA synaptic transmission in latent inflammatory pain model would not be the same in female mice. 2) The effect of PGB on the left CeA neurons. In this study, we demonstrated the effects of PGB in the right CeA. It is now well acknowledged that the right and left amygdala plays distinct roles in the pain-associated phenotypes (Allen et al., 2020, Miyazawa et al., 2018, Sadler et al., 2017). These issues should be addressed in future studies. 3) Translational study on “chronic” pain. One of the major issues of the translational study of pain is the definition of chronic pain in animal models. Animal models of pain with a duration of longer than three months as defined for human patients are not so widely used yet. It should be noted that the model we used (8 h post-formalin) only represents the process of the plastic shift of the neural network at a very early period. However, on the other hand, the changes we described in this study are not the direct acute response to the noxious stimulation but contain shifts towards a consolidated chronic pain-like state through neuronal plasticity of the nociceptive systems. Indeed, we have demonstrated changes in the whole brain network activity in the same intraplantar formalin injection model of mice using high-magnetic field small-animal MRI (Arimura et al., 2019). We have shown that the activated brain regions spread to many limbic, mesencephalic and brainstem areas from 2 h to 6 h post-formalin injection, as a consequence of the amygdala activation (Arimura et al., 2019). It should be kept in mind that the findings presented here may not necessarily represent what happens in patients with chronic pain taking PGB.

## Conclusion

PGB inhibited BLA-to-CeA transmission, but not LPB-to-CeA transmission, in the brain from mice with inflammatory pain. Such pathway dependence might partly define the spectrum and also the effectiveness of PGB in treating the cognito-affective aspect of pain.

### CRediT authorship contribution statement

**Sumii Yamamoto:** Funding acquisition. **Yukari Takahashi:** Funding acquisition. **Fusao Kato:** Funding acquisition.

## Declaration of Competing Interest

The authors declare the following financial interests/personal relationships which may be considered as potential competing interests: Fusao Kato is a recipient of the collaborative study on the gabapentinoid effects with Daiichi-Sankyo Co. Ltd.
